# Complete Mitochondrial Genome of *Malenka flexura* (Plecoptera: Nemouridae) and Phylogenetic Analysis

**DOI:** 10.3390/genes13050911

**Published:** 2022-05-19

**Authors:** Jinjun Cao, Xuan Guo, Caiyue Guo, Xuan Wang, Ying Wang, Fengming Yan

**Affiliations:** 1 College of Plant Protection, Henan Agricultural University, Zhengzhou 450002, China; cjj1986108@163.com; 2 Postdoctoral Research Base, Henan Institute of Science and Technology, Xinxiang 453003, China; guoxuan1999@163.com (X.G.); gcy159365@163.com (C.G.); wx1292546865@163.com (X.W.)

**Keywords:** mitochondrial genome, Amphinemurinae, *Malenka*, phylogeny

## Abstract

The genus-level relationships within the subfamily Amphinemurinae have been controversial, although attempts have been made based on morphology and limited molecular data. With the establishment of two new genera, the phylogenetic relationships within Amphinemurinae should be re-examined. In this study, the complete mitochondrial genome (mitogenome) of *Malenka flexura* of the genus *Malenka* was firstly sequenced and analyzed. The phylogeny of Amphinemurinae was also reconstructed using 13 proteincoding genes (PCGs) from previously published stoneflies. This mitogenome was 15,744 bp long and encoded the typical 37 genes, as well as a putative control region. The gene arrangement of *M. flexura* mitogenome is identical with the putative ancestral mitogenome in *Drosophila yakuba*. Most PCGs used standard ATN as start codons and TAA/TAG as termination codons. All tRNA genes exhibited the typical cloverleaf secondary structure, except for *tRNA^Ser(AGN)^*, whose dihydrouridine (DHU) arm was lacking. Some structural elements in the control region were founded, such as tandem repeat regions, stemloop structures, polyN stretch and microsatellite structure, etc. Phylogenetic analyses of sequenced Amphinemurinae mitogenomes unsupported the sister relationship of *Amphinemura* and *Malenka*. Finally, the phylogenetic analyses inferred a relationship within Amphinemurinae: *Amphinemura* + (*Malenka* + (*Protonemura* + (*Indonemoura* + (*Sphaeronemoura* + *Mesonemoura*)))).

## 1. Introduction

The mitochondrial genome (mitogenome) is a complete and relatively independent organelle genome, which contains complete genetic information from molecular sequence to gene structure [1,2]. Insect mitogenome typically constitutes 14–20 kb circular DNA molecules. It encodes 13 protein-coding genes (PCGs), two ribosomal genes (rRNAs), and 22 transfer RNA genes (tRNAs) [1,3]. It also has an A + T-rich region (or control region, CR) that regulates the transcription and replication of the mitogenome [3]. In recent years, the mitogenome has become a major resource for investigating biogeography, species evolution, population genetics structure, and phylogeny of different classification elements [4,5,6,7], because of its small genome size, rare recombination, rapid mutation rate, maternal inheritance, and conserved gene content [1,8].

Stoneflies (Insecta: Plecoptera) are a group of hemimetabolous aquatic insects that are distributed around the world except for Antarctica [9,10]. Currently, over 4000 extant species are described in the order Plecoptera, which are divided into 17 families [9,10,11]. Stoneflies are most commonly associated with clean, cool running water and cool, wet terrestrial environments [10]. The nymphs congregate in riffle areas of streams with an abundance of boulders, gravel, snags, and piled leaves. It is commonly recognized that they could be used as biological indicators of water quality [12].

Nemouridae is one of the largest Plecoptera families, with over 400 species distributed across the nearctic, palearctic, and oriental regions [13]. The nymphs are distinguished by their broad, bristly bodies and divergent wing pads. They can be found in a wide range of streams, but smaller creeks and spring runs are probably the most diverse [9]. Baumann considered Amphinemurinae as a new subfamily of Nemouridae based on the number of lobes on parprocts, and divided this subfamily into five genera, *Amphinemura*, *Indonemoura*, *Malenka*, *Mesonemoura* and *Protonemura* [13]. Recently, *Sphaeronemoura* and *Tominemoura* were proposed as two new genera in this subfamily [14,15]. Therefore, the Amphinemurinae presently includes seven described genera worldwide.

So far, Baumann’s morphological analyses of the phylogenetic relationships within the Nemouridae are thought to be the most comprehensive [13]. At the subfamily level, the Amphinemurinae is recognized as monophyletic, and the relationship within this subfamily was recovered as (*Amphinemura* + *Malenka*) + (*Protonemura* + (*Indonemoura* + *Mesonemoura*)). Early molecular phylogeny of stoneflies has been studied using a single nucleotide sequence [16] and six molecular markers [17]. Both studies used fewer genera of this subfamily, and the results did not support the sister group relationship of *Amphinemura* and *Malenka*. Recently, phylogenies for Amphinemurinae fauna were proposed using mitochondrial genomic data, and those results showed that the positions of *Mesonemoura*, *Indonemoura* and *Protonemura* were similar to the traditional morphology-based results [18,19,20]. However, only sequences from five genera of Amphinemurinae species (*Amphinemura*, *Protonemura*, *Indonemoura*, *Mesonemoura* and *Sphaeronemoura*) were included in those previous studies.

To better resolve the phylogenetic relationship within this subfamily, more Amphinemurinae mitogenomes, particularly those from *Malenka* and *Tominemoura* species, should be obtained. Because the genus *Tominemoura* has only one described species (*Tominemoura trilari*), and this species is only found in Sabah, Malaya, the specimen of *T. trilari* was unable to be obtained. In this study, one complete mitogenome of genera *Malenka* (*Malenka flexura*) was sequenced, and its nucleotide compositions, codon usage and RNA structures were analyzed. Finally, the phylogenetic analyses of Amphinemurinae were performed based on the nucleotide sequences of available stonefly mitogenomes. The aim of this research is to improve the understanding of the phylogeny of Amphinemurinae.

## 2. Materials and Methods

### 2.1. Sample Collection and DNA Extraction

Wild specimens of *M. flexura* were collected from Albany in New York, USA, and the voucher specimen for this species (No. VHL-0135) was deposited in the Department of Entomology, Henan Institute of Science and Technology, China. Specimens used in this study were preserved in 100% ethanol and stored at −20 °C. Total genomic DNA was isolated from the thoracic muscle of adults using the DNeasy Blood & Tissue Kit (Qiagen, Hilden, Germany).

### 2.2. Sequencing and Bioinformatics Analyses

Illumina Hiseq 2500 with 500 cycles of paired-end sequencing (250 bp reads) was performed at Berry Genomics Co., Ltd., Beijing, China. The mitogenome of *M. flexura* was sequenced and amplified as described in previous studies [18,19,21,22,23]. Illumina sequence reads were assembled into contigs with Geneious 6.1.6 [24]. The tRNA genes were initially identified using the MITOS webservers [25]. The boundaries of PCGs and rRNA genes were identified by comparing with the homologous genes of other published stonefly species. Base composition, codon usage and the relative synonymous codon usage (RSCU) were calculated using MEGA 6.0 [26]. Composition skew analysis was performed using the AT-skew = [A − T]/[A + T] and GC-skew = [G − C]/[G + C] formulas [27]. Stem-loop structures in the control region were predicted by DNAMAN, and the tandem repeat units were identified using the Tandem Repeats Finder server (http://tandem.bu.edu/trf/trf.advanced.submit.html, accessed on 1 May 2022) [28].

### 2.3. Phylogenetic Analyses

A total of 17 nemourid species were used for the phylogenetic analysis, including 14 Amphinemurinae species and three outgroup species from the subfamily Nemourinae (Table 1). In total, 13 PCGs in the 17 species were aligned using the MAFFT algorithm [29] in the TranslatorX online platform [30]. The alignment of individual genes was concatenated together to make the PCG dataset (including 13 PCGs) after removing ambiguously aligned positions.

Phylogenetic trees were reconstructed based on the PCG dataset under the maximum likelihood (ML) and Bayesian inference (BI) methods. According to the Akaike information criterion (AIC), the best-fit model GTR + I+G for the dataset was determined using ModelFinder [31]. ML analysis was performed using IQ-TREE Web Server [31] with 10,000 bootstrap replicates. BI analysis was carried out with MrBayes 3.2.6 [32] under the following conditions: 10 million generations with sampling every 1000 generations, four independent Markov chains, and a burn-in of 25% trees.

## 3. Results and Discussion

### 3.1. Mitogenome Organization and Base Composition

**Figure 1:**
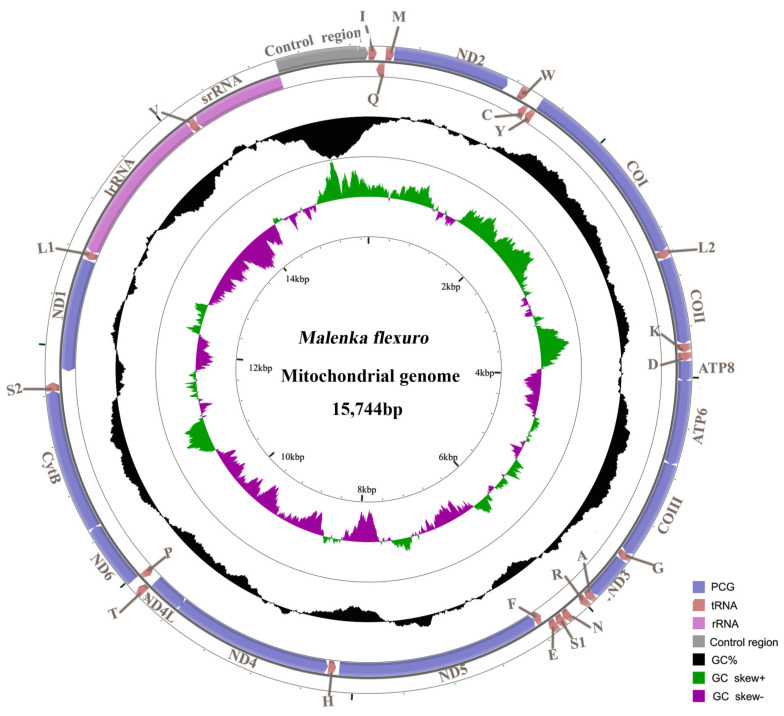
Map of the mitogenome of *M. flexura*. tRNA genes are labeled using abbreviations. The content of Guanine and Cytosine nucleotides (GC content) is plotted as the deviation from the average GC content of the entire sequence. GC skew is plotted as the deviation from the average GC skew of the entire sequence.

The complete mitogenome of *M. flexura* is 15,744 bp in length (GenBank accession number ON411527; Figure 1), which is consistent with other sequenced Amphinemurinae species [18]. It is a double-stranded circular molecule, including 13 PCGs, 22 tRNA genes, 2 rRNA genes and a large non-coding region (control region) (Table 2 and Figure 1). The gene order of the *M. flexura* mitogenome is identical to other sequenced stoneflies and the model insect, *Drosophila yakuba* [33]. There are 51 overlapping nucleotides distributed in 13 gene junctions; the *tRNA^Trp^*/*tRNA^Cys^* and *tRNA^Tyr^*/*COI* gene junctions possess the longest overlap (8 bp). The *ATP8*/*ATP6* and *ND4*/*ND4L* gene junctions overlap seven nucleotides (ATGNTAA), and are often found across the Metazoa [34,35]. Except for the large non-coding region, there are 225 nucleotides dispersed in 11 intergenic spacers, ranging in size from 1 to 111 bp. (Table 2).

The overall nucleotide composition of the *M. flexura* mitogenome is 32.3% A, 18.8% G, 36.3% T and 12.5% C, respectively (Table 3). The A + T content of the whole mitogenome, PCGs, tRNAs, rRNAs and the control region is 68.6%, 66.6%, 70.9%, 71.9% and 85.2% (Table 3). Therefore, the nucleotide composition of the *M. flexura* mitogenome is biased toward A and T nucleotides, and the control region is usually considered the most A + T rich in stonefly mitogenomes [18,19,20,21,22,23].

The nucleotide composition of metazoan mitogenomes usually has a clear strand bias [36,37], which can be measured as AT- and GC-skews [27]. In this study, the *M. flexura* mitogenome shows a negative AT-skew and a positive GC-skew (Table 3), revealing a bias in the use of T and G nucleotides. For the J-strand, most insect mitogenomes show a positive AT-skew and negative GC-skew [38], while results of this study show a negative AT-skew of PCGs and a positive GC-skew of tRNA genes. Like the *M. flexura* mitogenome, the strand bias of some other stonefly mitogenomes is also inconsistent with that of most other insects (positive AT skew and negative GC skew for the J-strand) [18,21,39,40]. The balance between mutational and selection pressures during replication and transcription may cause nucleotide compositional asymmetries, which might serve as a possible signal for replication orientation and gene direction [27,38].

### 3.2. Protein-Coding Genes and Codon Usage

Most PCGs of *M. flexura* use ATN as the start codon, such as ATT (4 PCGs), and ATG (7 PCGs). However, two exceptions, *ND5* and *ND1* genes, initiate with GTG and TTG as a start codon, respectively (Table 2). The use of these two nonstandard start codons is also found in other Amphinemurinae species [18]. In some species, TTG is also employed to shorten intergenic spacer and avoid gene overlap [41,42]. Eight PCGs (*ND2*, *COI*, *ATP8*, *ATP6*, *COIII*, *ND4*, *ND4L* and *ND6*) terminate with the stop codon TAA, three PCGs (*ND3*, *CytB* and *ND1*) terminate with TAG, and two PCGs (*COII* and *ND5*) end with incomplete stop codon T (Table 2). The use of an incomplete stop codon (T) is common in stoneflies [18,20,21,22,23,39,40,43] and animal mitogenomes [3], and can form a complete TAA terminal signal by post-transcriptional polyadenylation [44,45].

**Figure 2:**
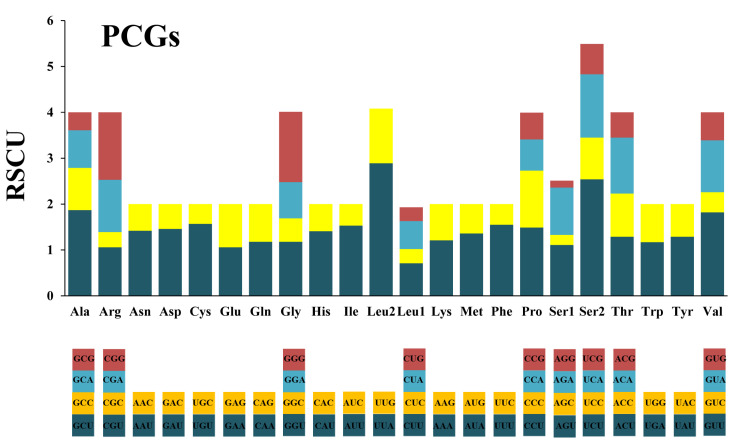
The relative synonymous codon usage (RSCU) in the mitogenome of *M. flexura*. PCGs represent protein-coding genes.

The influence of a strong biased codon usage is reflected by the relative synonymous codon usage (RSCU) [46]. The result shows that both two-fold and four-fold degenerate codons are preferable over codons ending with A or U (Figure 2). Another five prevalent AT-rich codons (TTA, ATT, TTT, ATA and AAT) also contribute to the compositional biases for AT (Table 4).

### 3.3. Transfer and Ribosomal RNA Genes

The lengths of tRNAs are ranged from 63 bp to 71 bp (Table 2). All tRNA genes exhibit the typical cloverleaf secondary structure, except for *tRNA^Ser(AGN)^*, whose dihydrouridine (DHU) arm is lacking (Figure S1). According to the secondary structure of *M. flexura* tRNA genes, there are 42 unmatched base pairs in these tRNAs. Thirty-four of these are weak G-U pairs, which are in acceptor arms (9 bp), DHU arms (10 bp), anticodon arms (12 bp), and TΨC arms (3 bp). The remaining are U-U (1 bp), A-G (2 bp), U-C (3 bp) and A-C (2 bp) mismatches (Figure S1).

The large rRNA subunit gene (*lrRNA*) is 1339 bp long, with an A + T content of 73.6%, whereas the small rRNA subunit gene (*srRNA*) is 790 bp long, with an A + T content of 68.9%. The *lrRNA* and *srRNA* genes present in the *M. flexura* mitogenome are located between *tRNA^Leu(CUN)^* and *tRNA^Val^*, and between *tRNA^Val^* and the control region, respectively. Results of the rRNAs secondary structures show that *lrRNA* and *srRNA* have five (I–II, IV–VI, with domain III absent) and three (I–III) structural domains, respectively (Figures S2 and S3). Both *lrRNA* and *srRNA* have characteristics that are similar to those found in most published plecopteran species [18,20,21,22,23,39].

### 3.4. The Control Region

The control region contains essential elements involved in the initiation of replication and transcription of the mitogenome [47]. The control region of *M. flexura* is 735 bp in length and is located between *srRNA* and *tRNA^Ile^* (Figure 1 and Table 2). It contains the highest A + T content (85.2%) in the entire mitogenome.

**Figure 3:**
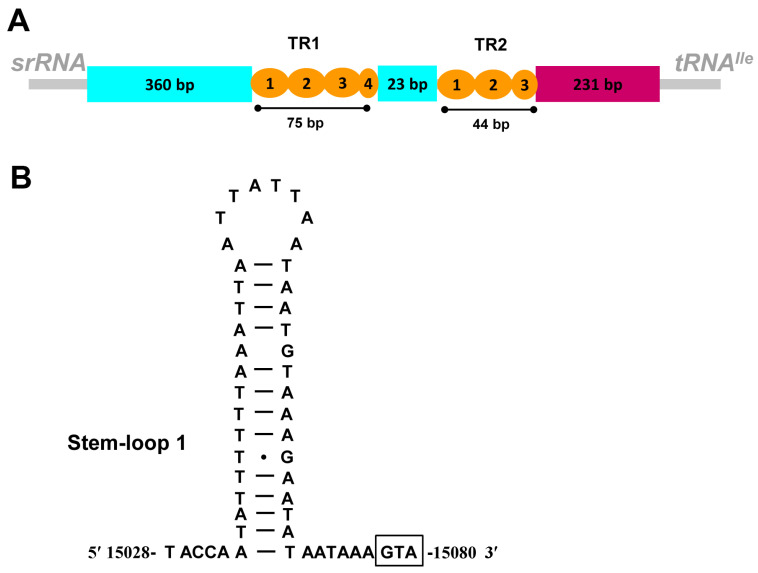
Control region of the *M. flexura* mitogenome. (**A**) Structure elements found in the control region of *M. flexura*. (**B**) Putative stem-loop structures found in the control region of *M. flexura*.

The control region of *M. flexura* can be divided into five parts: (1) a 360 bp leading sequence adjacent to *srRNA* composed of a stem-loop structure; (2) a 75 bp tandemly repeated sequence block consisting of three complete and one incomplete tandem repeat units; (3) a 23 bp region; (4) a 44 bp region including two complete and one incomplete tandem repeats units; (5) a 231 bp region at the end of the control region (Figure 3A).

One stem-loop (SL1, position: 15,028 bp–15,080 bp) structure is predicted in the control region (Figure 3B). The proposed SL structure with a 3′ flanking “G(A)nT” motif is not detected in SL1, but it is modified as “GTA”. The stem-loop structure in the control region is identified in many insects and it is thought to be the site of the initiation of secondary strand synthesis in *Drosophila* [48]. In addition, one microsatellite sequence (position: 15,376 bp–15,391 bp), (AT)8, is detected in the control region. Similar to other stoneflies mitogenomes [18,40,49], several poly-N stretch (≥7 bp) were also found near to the *tRNA^Ile^*, such as poly-T (9 bp, position: 15,668 bp–15,676 bp) and poly-A (9 bp, position: 15,574 bp–15,582 bp). Poly-T stretch is considered to be essential for the initiation of replication in insects [50].

### 3.5. Phylogenetic Relationships

**Figure 4:**
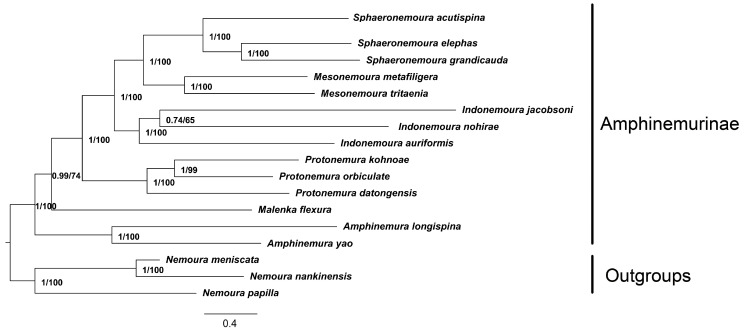
Mitochondrial phylogenetic relationships among 17 stoneflies. Bayesian inference and aximum likelihood analysis inferred from PCGs supported the same topological structure. Values at nodes are Bayesian posterior probabilities (BPPs) and ML bootstrap probabilities (BSPs). The tree was rooted with three outgroups.

In the present study, concatenated nucleotide sequences of 13 PCGs from 17 nemourid mitogenomes were used to reconstruct phylogenetic relationships by the BI and ML methods. Two methods generated the same tree topologies (Figure 4).

The monophyly of each genus is generally well supported (bootstrap probabilities (BSPs) ≥ 74; Bayesian posterior probabilities (BPPs) ≥ 0.99). In morphology, *Indonemoura*, *Mesonemoura*, *Protonemura* and *Sphaeronemoura* are similar to each other in having a sclerotized projection (tigellus) on the median lobe of the paraproct [13]. Although Shimizu and Sivec regarded the genus *Sphaeronemoura* not to be closely related to *Mesonemoura* [14], result of this study supports *Sphaeronemoura* as a sister group of *Mesonemoura*. This result is similar to that of Cao et al. (2019) [18]. In addition, the position of *Protonemura* and *Indonemoura* is consistent with the traditionally proposed relationships [13] and previous studies [18,19,20]. Moreover, the traditional morphology-based classification among Amphinemurinae was well supported [13].

*Malenka* is the sister genus of *Amphinemura* and is restricted to western North America. They usually have a distinct median notch with other genera of Amphinemurinae [13]. However, phylogenetic analyses in this study do not support the sister group relationship of *Amphinemura* and *Malenka*. This result is similar to that of Thomas et al. (2000) [16] and Terry (2003) [17], but differs from the morphological studies of Baumann (1975) [13]. More comprehensive sampling especially for those stoneflies from the *Malenka* is expected to better resolve the mitochondrial phylogeny of Amphinemurinae. Finally, the best-supported phylogenetic relationship found in this study is as follows: *Amphinemura* + (*Malenka* + (*Protonemura* + (*Indonemoura* + (*Sphaeronemoura* + *Mesonemoura*)))).

## 4. Conclusions

Currently, the position of five genera in Amphinemurinae has been resolved based on morphology. However, the results of early molecular studies differ from morphological results. With the establishment of two new genera (*Sphaeronemoura* and *Tominemoura*), the phylogenetic relationships within Amphinemurinae should be re-examined. In this study, one complete mitogenome from genus *Malenka*, in the subfamily Amphinemurinae, was presented. Its mitogenome organizations and phylogenetic relationships with the other species from Amphinemurinae were analyzed. The *M. flexura* mitogenome resulted in a DNA molecule with genomic features typical for insect mitogenomes, such as conserved gene order, gene content, nucleotide composition, codon usage of PCGs and RNA secondary structures. In addition, some structural elements were also found in the control region, such as tandem repeats regions, poly-N stretch, stem-loop structures, etc. The phylogenetic analyses indicated that within Amphinemurinae, *Amphinemura* and *Malenka* were not supported as a sister-group relationship. The relationship between *Amphinemura* and *Malenka* would be improved if a more comprehensive taxon sampling was used. Finally, phylogenetic analyses inferred a relationship within Amphinemurinae: *Amphinemura* + (*Malenka* + (*Protonemura* + (*Indonemoura* + (*Sphaeronemoura* + *Mesonemoura*)))).

## Figures and Tables

**Table 1:** General information of nemourid species used in this study.

Subfamily	Species	Number (bp)	Accession Number
Amphinemurinae	*Amphinemura longispina*	15,709	MH085446
	*Amphinemura yao*	15,876	MH085447
	*Indonemoura auriformis*	15,718	MN419915
	*Indonemoura jacobsoni*	15,642	MH085448
	*Indonemoura nohirae*	15,738	MH085449
	*Malenka flexura*	15,744	ON411527
	*Mesonemoura metafiligera*	15,739	MH085450
	*Mesonemoura tritaenia*	15,778	MH085451
	*Protonemura datongensis*	15,756	MT276842
	*Protonemura kohnoae*	15,707	MH085452
	*Protonemura orbiculata*	15,758	MH085453
	*Sphaeronemoura acutispina*	15,016	MH085455 *
	*Sphaeronemoura elephas*	15,846	MN944385
	*Sphaeronemoura grandicauda*	15,661	MH085454 *
Nemourinae (Outgroup)	*Nemoura meniscata*	15,895	MN944386
	*Nemoura nankinensis*	16,602	KY940360
	*Nemoura papilla*	15,774	MK290826

* Incomplete mitogenome sequence.

**Table 2:** Organization of the *M. flexura* mitochondrial genome.

Gene	Direction	Coordinates (bp)	Size (bp)	Anticodon or Start/Stop Codons	IGN (bp)
*tRNA^Ile^*	J	1–66	66	30–32 GAT	0
*tRNA^Gln^*	N	64–132	69	100–102 TTG	−3
*tRNA^Met^*	J	137–204	68	167–169 CAT	4
*ND2*	J	205–1239	1035	ATG/TAA	0
*tRNA^Trp^*	J	1247–1315	69	1277–1279 TCA	7
*tRNA^Cys^*	N	1308–1370	63	1339–1341 GCA	−8
*tRNA^Tyr^*	N	1377–1442	66	1409–1411 GTA	6
*COI*	J	1435–2979	1545	ATT/TAA	−8
*tRNA^Leu(UUR)^*	J	2975–3041	67	3017–3019 TAA	−5
*COII*	J	3045–3732	688	ATG/T-	3
*tRNA^Lys^*	J	3733–3803	71	3763–3765 CTT	0
*tRNA^Asp^*	J	3803–3870	68	3832–3834 GTC	−1
*ATP8*	J	3871–4029	159	ATT/TAA	0
*ATP6*	J	4023–4700	678	ATG/TAA	−7
*COIII*	J	4700–5488	789	ATG/TAA	−1
*tRNA^Gly^*	J	5488–5553	66	5517–5519 TCC	−1
*ND3*	J	5554–5907	354	ATT/TAG	3
*tRNA^Ala^*	J	5906–5969	64	5935–5937 TGC	−2
*tRNA^Arg^*	J	5970–6032	63	5999–6001 TCG	0
*tRNA^Asn^*	J	6144–6209	66	6174–6172 GTT	111
*tRNA^Ser(AGN)^*	J	6209–6277	69	6235–6237GCT	−1
*tRNA^Glu^*	J	6277–6345	69	6307–6309 TTC	−1
*tRNA^Phe^*	N	6344–6408	65	6376–6378 GAA	−2
*ND5*	N	6409–8143	1735	GTG/T-	0
*tRNA^His^*	N	8144–8209	66	8177–8179 GTG	0
*ND4*	N	8213–9553	1341	ATG/TAA	3
*ND4L*	N	9547–9843	297	ATG/TAA	−7
*tRNA^Thr^*	J	9846–9911	66	9877–9879 TGT	2
*tRNA^Pro^*	N	9911–9975	65	9943–9945 TGG	−1
*ND6*	J	9977–10,501	525	ATT/TAA	0
*CytB*	J	10,501–11,637	1137	ATG/TAG	−1
*tRNA^Ser(UCN)^*	J	11,636–11,705	70	11,667–11,669 TGA	−2
*ND1*	N	11,792–12,742	951	TTG/TAG	86
*tRNA^Leu(CUN)^*	N	12,744–12,809	66	12,778–12,780TAG	1
*lrRNA*	N	12,810–14,148	1339		0
*tRNA^Val^*	N	14,149–14,219	71	14,184–14,1186 TAC	0
*srRNA*	N	14,220–15,009	790		0
CR		15,010–15,744	735		0

CR—control region; IGN—intergenic nucleotides; J—majority strand; N—minority strand.

**Table 3:** The nucleotide composition of the *M. flexura* mitogenome.

Feature	Proportion of Nucleotides (%)					AT Skew	GC Skew
	T	C	A	G	A + T		
Whole mitogenome	36.3	12.5	32.3	18.8	68.6	−0.059	0.200
Protein-coding genes	39.3	17.1	27.3	16.3	66.6	−0.180	−0.022
Protein-coding genes J-strand	35.6	20.7	29.1	14.7	64.7	−0.101	−0.171
Protein-coding genes N-strand	45.1	11.4	24.4	19.1	69.6	−0.297	0.2552
tRNA genes	35.5	12.2	35.4	16.9	70.9	−0.001	0.160
tRNA genes J-strand	34.9	13.9	36.0	15.2	70.9	0.015	0.044
tRNA genes N-strand	36.5	9.2	34.5	19.9	71.0	−0.029	0.368
rRNA genes	39.0	10.1	32.9	18.1	71.9	−0.084	0.285
*lrRNA*	40.4	9.0	33.2	17.4	73.6	−0.097	0.320
*srRNA*	36.5	12.0	32.3	19.2	68.9	−0.061	0.231
Control region	43.6	8.1	41.6	6.7	85.2	−0.023	−0.100

**Table 4:** Codon number in the *M. flexura* mitochondrial PCGs.

Codon	Count	Codon	Count	Codon	Count	Codon	Count
UUU(F)	270	UCU(S)	95	UAU(Y)	109	UGU(C)	34
UUC(F)	51	UCC(S)	31	UAC(Y)	48	UGC(C)	8
UUA(L)	364	UCA(S)	74	UAA(*)	0	UGA(W)	87
UUG(L)	46	UCG(S)	12	UAG(*)	0	UGG(W)	19
CUU(L)	102	CCU(P)	63	CAU(H)	58	CGU(R)	14
CUC(L)	31	CCC(P)	38	CAC(H)	25	CGC(R)	7
CUA(L)	71	CCA(P)	42	CAA(Q)	64	CGA(R)	32
CUG(L)	12	CCG(P)	9	CAG(Q)	117	CGG(R)	7
AUU(I)	271	ACU(T)	87	AAU(N)	127	AGU(S)	45
AUC(I)	41	ACC(T)	27	AAC(N)	23	AGC(S)	17
AUA(I)	153	ACA(T)	75	AAA(K)	52	AGA(S)	63
AUG(M)	38	ACG(T)	12	AAG(K)	20	AGG(S)	3
GUU(V)	105	GCU(A)	92	GAU(D)	52	GGU(G)	53
GUC(V)	28	GCC(A)	38	GAC(D)	18	GGC(G)	34
GUA(V)	77	GCA(A)	54	GAA(E)	55	GGA(G)	83
GUG(V)	23	GCG(A)	24	GAG(E)	24	GGG(G)	79

* represent the stop codons.

## Data Availability

The data that support the findings of this study are deposited in GenBank with accession number ON411527. The data are available from the corresponding authors upon reasonable request.

## Supplementary Materials

The following supporting information can be downloaded at: https://www.mdpi.com/article/10.3390/genes13050911/s1, Figure S1: Predicted secondary structure of 22 tRNAs in *M. flexura* mitogenome; Figure S2: Predicted secondary structure of the *lrRNA* gene in *M. flexura* mitogenome; Figure S3: Predicted secondary structure of the *srRNA* gene in *M. flexura* mitogenome.
