# Supplementary material for: Complete Mitochondrial Genome of Malenka flexura (Plecoptera: Nemouridae) and Phylogenetic Analysis
Source: Genes (Basel). 2022 May 19;13(5):911. doi: 10.3390/genes13050911 (PMC9142110; doi:10.3390/genes13050911)
Supplement: Supplementary file 1 [file genes-13-00911-s001.zip › genes-1737114-SI.pdf]

Supplementary Materials

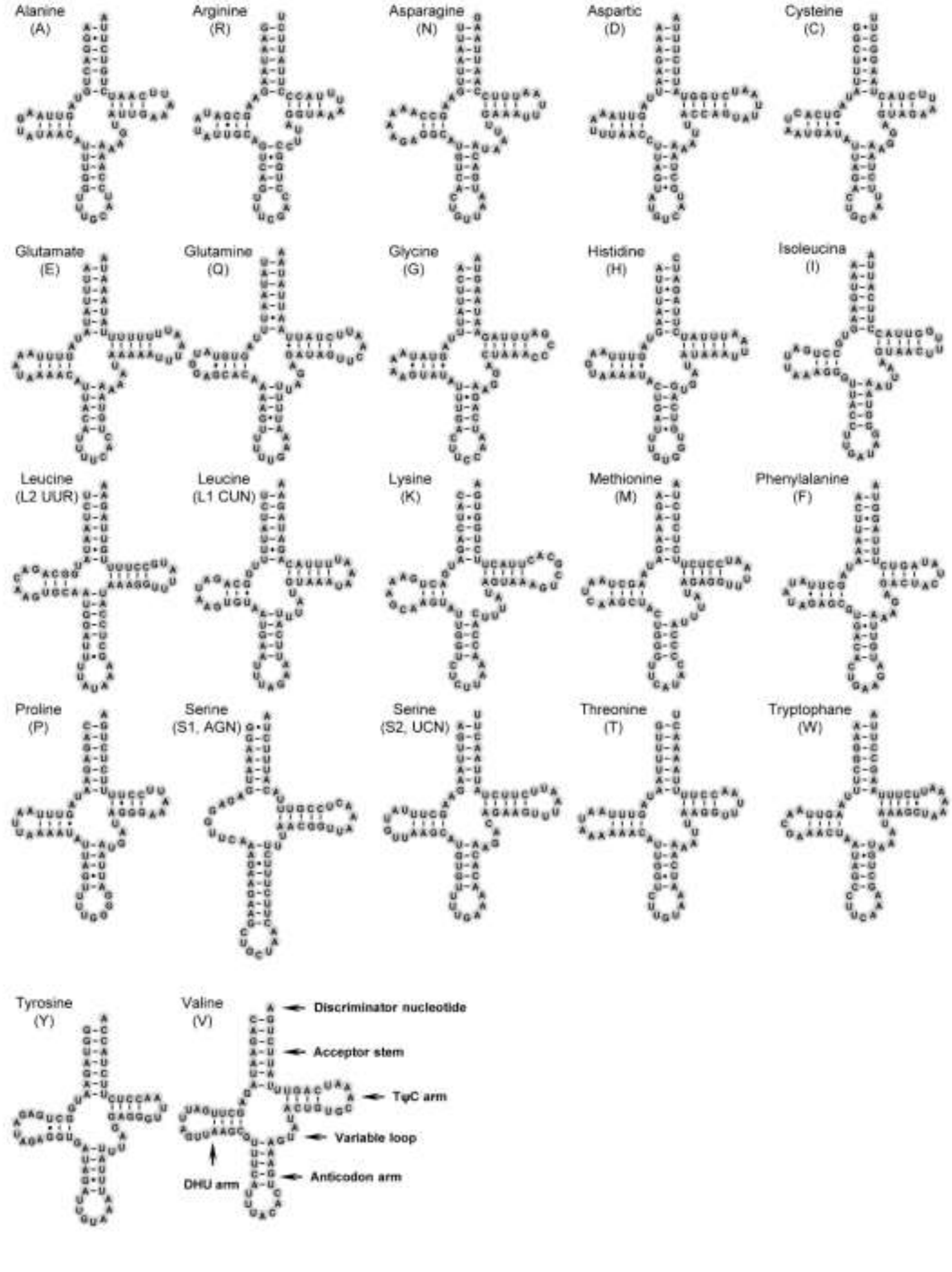

Figure S1. Predicted secondary structure of 22 tRNAs in *M. flexura* mitogenome.

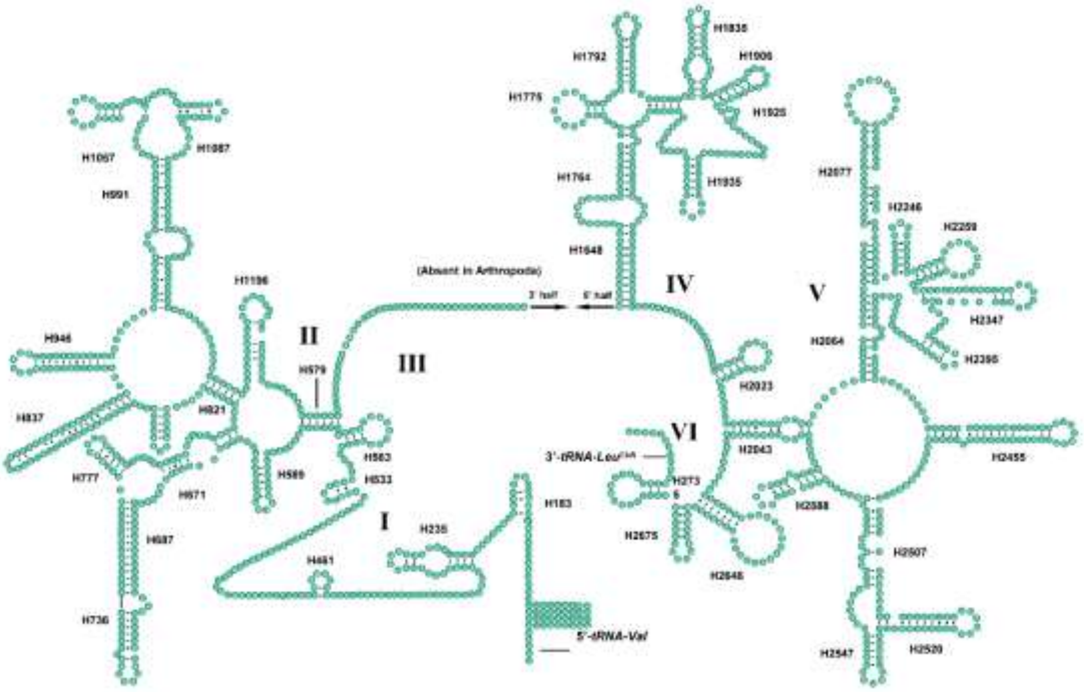

Figure S2. Predicted secondary structure of the *lrRNA* gene in *M. flexura* mitogenome.

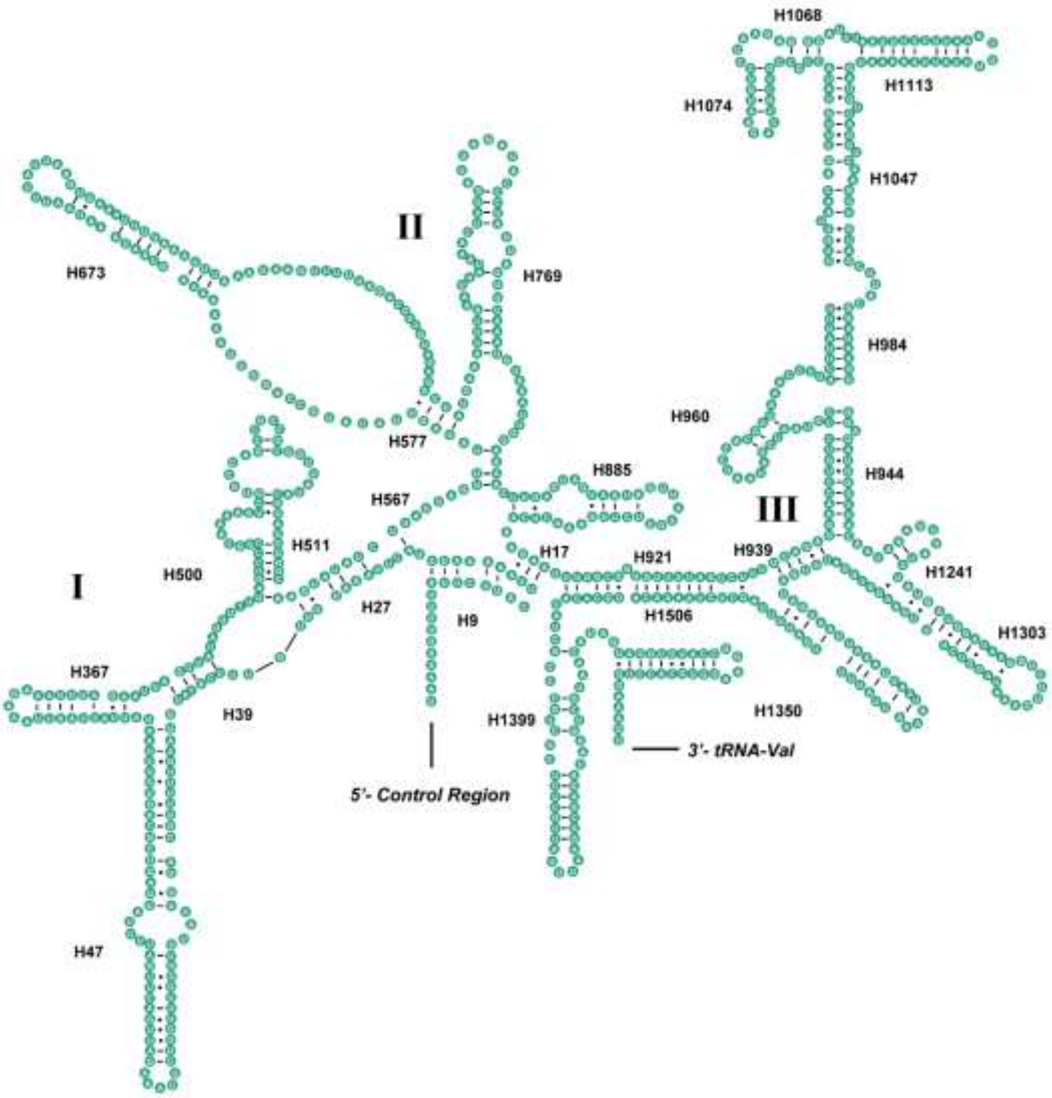

Figure S3. Predicted secondary structure of the *srRNA* gene in *M. flexura* mitogenome.
